# Fast Multichannel Inverse Design through Augmented Partial Factorization

**DOI:** 10.1021/acsphotonics.3c00911

**Published:** 2024-01-04

**Authors:** Shiyu Li, Ho-Chun Lin, Chia Wei Hsu

**Affiliations:** Ming Hsieh Department of Electrical and Computer Engineering, University of Southern California, Los Angeles, California 90089, United States

**Keywords:** inverse design, topology optimization, augmented partial factorization, metasurface, wide field of view

## Abstract

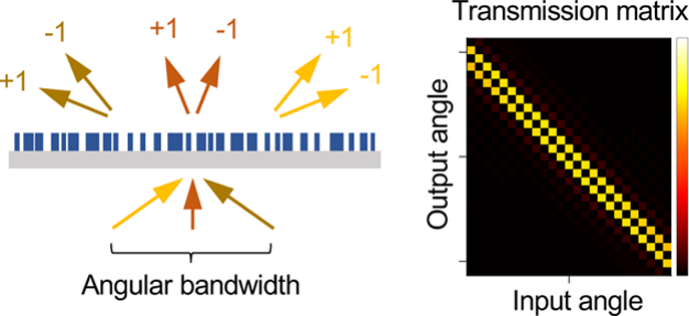

Computer-automated design and discovery have led to high-performance nanophotonic devices with diverse functionalities. However, massively multichannel systems such as metasurfaces controlling many incident angles and photonic-circuit components coupling many waveguide modes still present a challenge. Conventional methods require *M*_in_ forward simulations and *M*_in_ adjoint simulations—2*M*_in_ simulations in total—to compute the objective function and its gradient for a design involving the response to *M*_in_ input channels. Here, we develop a formalism that uses the recently proposed augmented partial factorization method to obtain both the objective function and its gradient for a massively multichannel system in a single or a few simulations, achieving over 2 orders of magnitude speedup and reduced memory usage. We use this method to inverse design a metasurface beam splitter that separates the incident light to the target diffraction orders for all incident angles of interest, a key component of the dot projector for 3D sensing. This formalism enables efficient inverse design for a wide range of multichannel optical systems.

## Introduction

Nanoengineered photonic devices can realize versatile and high-performance functionalities in a compact footprint, expanding the limited scope of conventional optical components. Computer-automated inverse design^1–12^ can search a high-dimensional parameter space to discover optimal structures that outperform manual designs or realize new functionalities. With an inverse design, the photonic structure is updated iteratively to optimize an objective function *f* that encapsulates the desired properties. Given the many parameters **p** = {*p*_*k*_} used to parametrize the design, efficient optimization typically requires gradient **∇**_**p**_*f* of the objective function with respect to all parameters. The computational efficiency is a critical consideration since full-wave simulations are necessary to model the complex light–matter interactions at the subwavelength scale, and numerous simulations are needed for the many iterations of the search process.

When the objective function *f* involves the response to just one input (*e.g.*, one incident angle) or one output (*e.g.*, one outgoing angle), the adjoint method can compute the complete gradient **∇**_**p**_*f* using only one forward and one adjoint simulations.^3,6–8^ However, when *f* involves *M*_in_ ≫ 1 inputs, the adjoint method requires 2*M*_in_ simulations (*M*_in_ forward, *M*_in_ adjoint).^13^ This prohibits the inverse design of large multichannel systems. There are many such multichannel systems including photonic circuits^14^ and aperiodic meta-structures for applications in wide-field-of-view lenses,^13,15–20^ beam combiners,^21,22^ angle-multiplexed holograms,^23,24^ concentrators,^15,25^ thermal emission control,^26–28^ image processing,^29–35^ optical computing,^36,37^ space compression,^38–40^*etc.* The inverse design of these systems remains challenging.

We recently proposed the “augmented partial factorization” (APF) method, which can solve multi-input electromagnetic forward problems in one shot, offering substantial speed-up and memory usage reduction compared to existing methods.^41^ However, the inverse problem was unsolved since the formalism of ref (41) only addresses forward response problems and does not yield the gradient. Here, we develop a formalism that generalizes APF to enable efficient gradient computation for multi-input problems. We can obtain both *f* and **∇**_**p**_*f* in a single or a few computations without a loop over the individual input channels. For a 1 mm wide metasurface with 2400 inputs, APF achieves ∼150 times speed-up and ∼30% memory usage reduction compared to the conventional adjoint method. As an example problem, we inverse design a metasurface beam splitter that splits the incident light equally into the ±1 diffraction orders over an incident angular range of 60°.

## Results and Discussion

### Multi-input Gradient Computation Using APF

**Figure 1:**
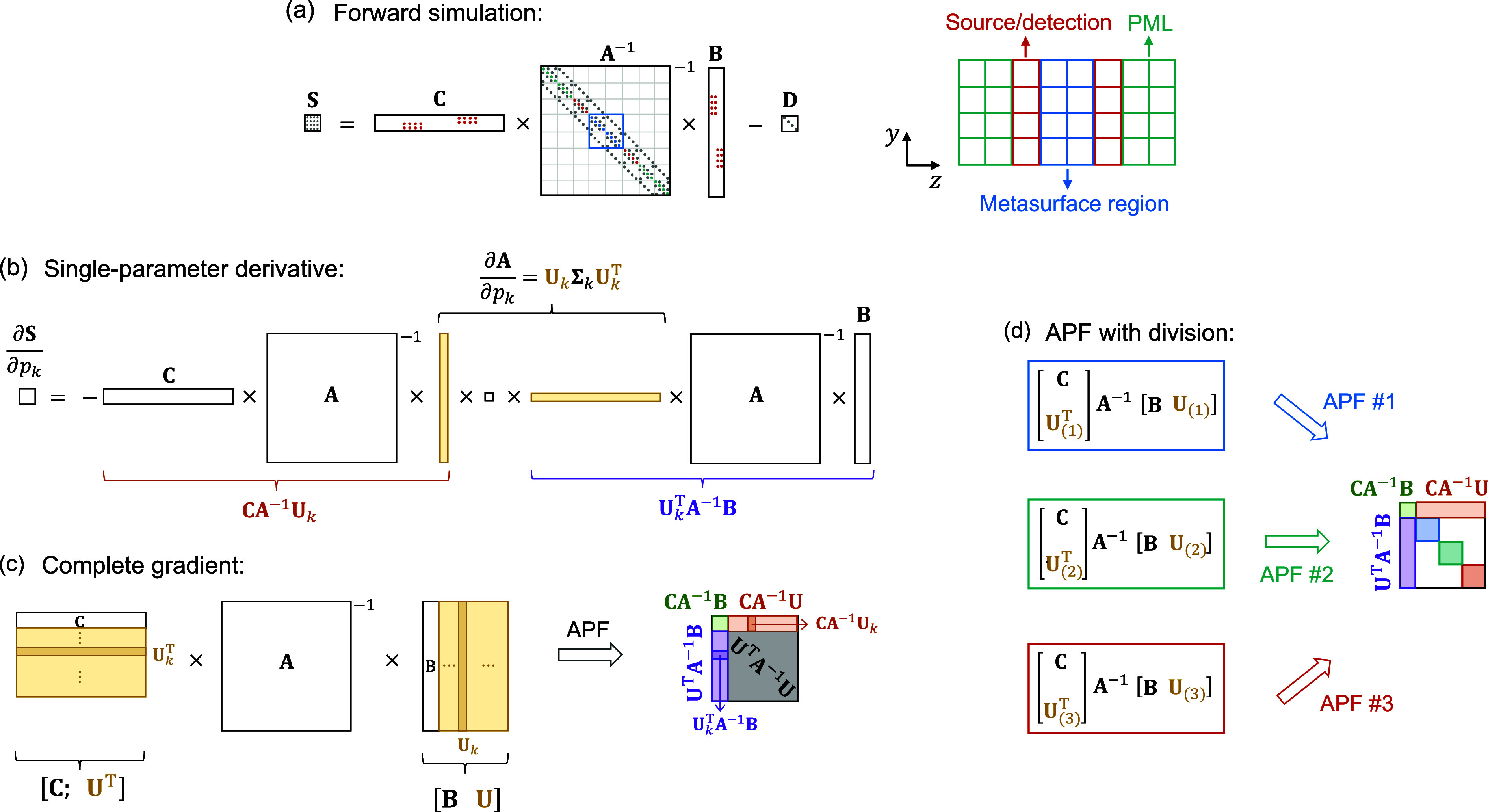
Schematics of using augmented partial factorization (APF) for (a) forward simulation and (b–d) gradient computation. (a) Illustration of **S = CA**^–1^**B – D**, where the multichannel response is encapsulated in a generalized scattering matrix **S** given in terms of the discretized Maxwell operator matrix **A**, the source profiles **B**, the projection profiles **C**, and the baseline **D** from the incident field. Dots represent nonzero elements of these sparse matrices, with colors corresponding to different regions of the simulation domain. PML: perfectly matched layer. (b) Illustration of eqs 2 and 3 for a single derivative: after decomposing ∂**A**/∂*p*_*k*_ into **U**_*k*_Σ_*k*_**U**_*k*_^T^ and regrouping the terms, **CA**^–1^**U**_*k*_ and **U**_*k*_^T^**A**^–1^**B** can be computed to yield ∂**S**/∂*p*_*k*_. (c) Illustration of eq 4 for the full gradient: by combining **U**_*k*_ for all parameters {*p*_*k*_} and augmenting them to **B** and **C**, a single APF computation can yield the complete gradient with respect to all parameters. (d) By dividing **U** and performing *N*_sub_ separate APF computations (*N*_sub_ = 3 in this illustration), we can obtain the same **CA**^–1^**B**, **CA**^–1^**U**, and **U**^T^**A**^–1^**B** with less computing time and memory by evaluating only 1/*N*_sub_ of the unnecessary matrix **U**^T^**A**^–1^**U** and by storing smaller matrices **CA**^–1^**U**_(*n*)_ and **U**_(*n*)_^T^**A**^–1^**B** in memory.

The novelty of the APF method lies in encapsulating the linear response of the multichannel system in a generalized scattering matrix **S = CA**^–1^**B – D** and then computing the entire **S** in a single shot through the partial factorization of an augmented matrix **K** = [**A**, **B**; **C**, **D**] that yields its Schur complement −**CA**^–1^**B**.^41^ Doing so bypasses the evaluation of volumetric field profile **A**^–1^**B**. Here, the *S*_*nm*_ element of the *M*_out_ × *M*_in_ matrix **S** is the field amplitude in the output channel *n* given an input in channel *m* at frequency ω. Matrix **A** is the discretized Maxwell differential operator −(ω/*c*)^2^ε_r_(**r**) + ∇ × ∇× of the structure defined by its relative permittivity profile ε_r_(**r**), the *M*_in_ columns of matrix **B** contain the *M*_in_ distinct input source profiles, the *M*_out_ rows of matrix **C** contain the *M*_out_ output projection profiles, and matrix **D** subtracts the baseline contribution from the incident field; they are schematically shown in Figure 1a. When the number of nonzero elements in matrices **B**, **C**, and **S** are less than the number of nonzero elements in matrix **A**, the single-shot computing time and memory usage of APF is roughly independent of how many input and output channels there are.^41^ In the following, we use APF with finite-difference discretization implemented in our open-source software MESTI,^42^ using the MUMPS package for factorization.^43^

Here, we derive a general formulation such that the gradient of any multichannel objective function can also be computed in a single shot regardless of the number *M*_in_ of input channels. We use vector **p** = [*p*_1_, ..., *p*_*N*_*p*__] to denote the *N*_*p*_ real-valued variables parametrizing the photonic design; in the example later, {*p*_*k*_} will be the edge positions of the ridges of a metasurface. The objective function *f* (also called the figure of merit, FoM) that evaluates the performance of the multichannel device is a function of the generalized scattering matrix **S** and the parameters **p**, namely *f*[**S**(**p**), **p**]. The gradient d*f*/d*p*_*k*_ we want follows from the chain rule as
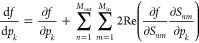
1Note that *S*_*nm*_ is complex-valued, and ∂*f*/∂*S*_*nm*_ is a Wirtinger derivative. Both ∂*f*/∂*p*_*k*_ and ∂*f*/∂*S*_*nm*_ can be calculated analytically given the definition of the objective function *f* for a specific problem. So, the simulations only need to evaluate the derivative of the scattering matrix, ∂*S*_*nm*_/∂*p*_*k*_.

The parameters {*p*_*k*_} modify the scattering matrix **S** by modifying the photonic structure ε_r_(**r**) in Maxwell operator **A**. Taking the derivative of **S = CA**^–1^**B – D** and using the identity ∂**A**^–1^/∂*p*_*k*_ = −**A**^–1^(∂**A**/∂*p*_*k*_)**A**^–1^, we get ∂**S**/∂*p*_*k*_ = −**CA**^–1^(∂**A**/∂*p*_*k*_)**A**^–1^**B**.

Here, we assume that matrices **B**, **C**, and **D** do not depend on {*p*_*k*_}; additional terms can be added if there is such dependence. This generalizes the adjoint method to multichannel systems: the columns of **A**^–1^**B** correspond to *M*_in_ forward problems, and the rows of **CA**^–1^ correspond to *M*_out_ adjoint problems. To recover the conventional adjoint method, one can substitute ∂**S**/∂*p*_*k*_ into eq 1 and sum over the output channels for each input, which converts the *M*_out_ adjoint problems to *M*_in_ adjoint problems (see Section S1); we do not do so with APF because doing so would require the forward problems to be solved prior to the adjoint ones, but for APF we want to solve the forward and the adjoint problems simultaneously.

A key observation is that the derivative ∂**A**/∂*p*_*k*_ above is a low-rank matrix since only a few elements of **A** depend on the parameter *p*_*k*_. For example, with 2D transverse magnetic (TM) waves at angular frequency ω, matrix **A** is the discretized version of −∇^2^ – (ω/*c*)^2^ε_r_(**r**), and ∂**A**/∂*p*_*k*_ is zero everywhere except for the few diagonal elements corresponding to pixels where the relative permittivity profile ε_r_(**r**) is modified by the parameter *p*_*k*_. Matrix ∂**A**/∂*p*_*k*_ is also symmetric due to reciprocity. Therefore, we can do a symmetric singular value decomposition
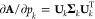
2where **Σ**_*k*_ is an *r*_*k*_-by-*r*_*k*_ diagonal matrix containing the singular values, *r*_*k*_ is the rank of ∂**A**/∂*p*_*k*_, the *r*_*k*_ columns of **U**_*k*_ are the left-singular vectors (which are real-valued and equal the right-singular vectors), and ^T^ stands for matrix transpose. These singular vectors are zero everywhere except at the pixels, where ε_r_(**r**) is modified by *p*_*k*_.

We then obtain the derivative of the scattering matrix with respect to the *k*th parameter
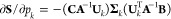
3as shown in Figure 1b. To obtain the complete gradient with respect to all *N*_*p*_ parameters {*p*_*k*_}, we combine the singular-vector matrices as **U** ≡ [**U**_1_, ..., **U**_*N*_*p*__], which has  columns. Computing **CA**^–1^**U** = [**CA**^–1^**U**_1_, ..., **CA**^–1^**U**_*N*_*p*__] and  with APF would yield the complete gradient through eqs 1–3 with just two APF computations. As shown in Figure 1c, we can further reduce from two APF computations to one by building new matrices **B̃** = [**B**, **U**] and  and using APF to compute
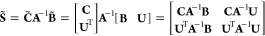
4Here, the matrix  is augmented with not only the original *M*_in_ input and *M*_out_ output channel profiles **B** and **C** but also *M*_*p*_ additional inputs/outputs being the singular vectors **U** and **U**^T^ from the design parameters {*p*_*k*_}. This way, a single-shot APF computation solves all of the *M*_in_ forward simulations (yielding the scattering matrix **S** from **CA**^–1^**B**) and also obtains the complete gradient (*i.e.*, ∂**S**/∂*p*_*k*_ and d*f*/d*p*_*k*_ for all *p*_*k*_, from **CA**^–1^**U** and **U**^T^**A**^–1^**B**) at the same time.

The computation time and memory usage of this APF-based multichannel gradient evaluation depend on *M*_*p*_, the number of pixels modified by the design variables. When the number of elements in matrix **S̃**, , is less than the number of nonzero elements in the Maxwell operator matrix **A**, the computing time and memory usage are independent of *M*_in_, *M*_out_, and *M*_*p*_, and we can include as many inputs/outputs and design variables as we want in a single APF computation. However when , the APF computing time and memory usage will grow linearly with ;^41^ this may be the case here since topology optimization often includes a large number of parameters. To mitigate this increase, we observe that the *M*_*p*_-by-*M*_*p*_ matrix **U**^T^**A**^–1^**U** in eq 4 (gray-shaded area in Figure 1c) is not needed for either the scattering matrix or the gradient. To improve the efficiency, we reduce how much of the large matrix **U**^T^**A**^–1^**U** we compute. As illustrated in Figure 1d, we do so by dividing the singular-vector matrix **U** into *N*_sub_ submatrices,  and separating the single APF computation into *N*_sub_ sub-APF computations, each operating on smaller matrices  and . This way, only 1/*N*_sub_ of the unnecessary matrix **U**^T^**A**^–1^**U** (areas shaded in red, green, and blue in Figure 1d) is computed, reducing the computing time and memory usage. To minimize memory, one can choose *N*_sub_ to reduce  of each sub-APF computation to the order of magnitude of nnz(**A**). One may merge the output of the *N*_sub_ computations to obtain  and similarly , but there is no such need: we can directly apply eq 3 onto **CA**^–1^**U**_(*n*)_ and **U**_(*n*)_^T^**A**^–1^**B** without merging them. By storing **CA**^–1^**U**_(*n*)_ instead of the entire **CA**^–1^**U**, we also further reduce memory usage further.

This formalism for computing the gradient of a multichannel objective function is very general. It applies to any dimension, polarization, discretization scheme, any type of input source profiles, and output projection profiles, with any objective function and any set of design variables.

As a concrete example, we consider full-wave modeling of the TM fields of a 1200-ridge aperiodic metasurface in 2D with mirror symmetry with respect to its central plane (*W* = 1200λ wide, *h* = 0.6λ thick, discretized with grid size Δ*x* = λ/40, where λ is the wavelength), computing its full transmission matrix with *M*_in_ = *M*_out_ = 2*W*/λ = 2400 plane-wave channels on each side and the gradient of the transmission matrix with respect to *N*_*p*_ = 1200 parameters being the edge positions of the ridges within half of the metasurface. Here, nnz(**A**) ≈ 1.6 × 10^7^, and *M*_*p*_ = 57,600. Compared to the conventional adjoint method, APF with *N*_sub_ = 15 reduces the gradient evaluation time from 1354 to 9.5 min and the memory usage from 10 to 7 GiB. Here, the conventional adjoint simulations are also performed with software MESTI,^42^ which is already optimized by utilizing the symmetry of the Maxwell operator matrix **A**, the sparsity of the input profiles, and the high-performance MUMPS package.^43^ All computations were run on a single core on an Intel Xeon 2640v4 node. Details are provided in Section S1 of the Supporting Information, Table S1 shows the breakdown of the computing times, and Figure S1 plots the dependence on *N*_sub_. We have made our gradient computation code open-source,^44^ including both the APF version and the conventional adjoint version.

### Inverse Design of a Broad-Angle Metasurface Beam Splitter

**Figure 2:**
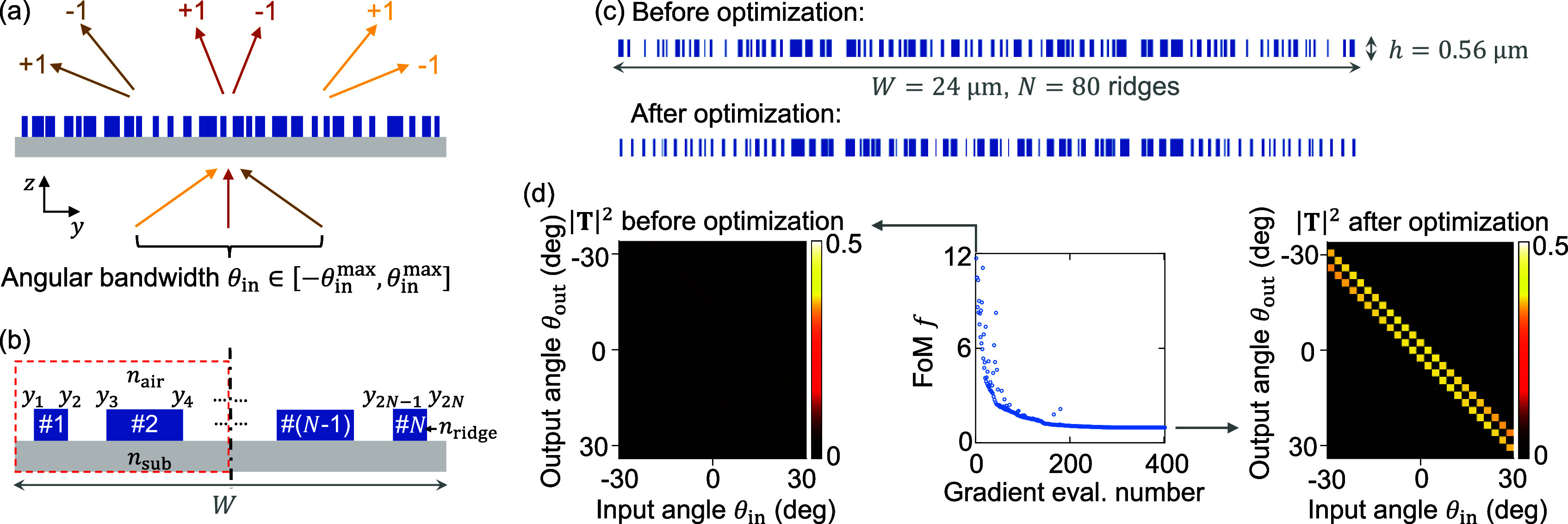
Inverse design of a broad-angle metasurface beam splitter. (a) Desired broad-angle beam-splitting response. (b) Parameter definition and mirror symmetry of the structure. (c) Metasurfaces before and after optimization. (d) Evolution of the FoM of eq 5 during optimization and the squared amplitude of the transmission matrices before and after optimization.

As an example, here, we inverse design a 2D metasurface beam splitter for TM polarization, composed of ridges with different widths, as shown in Figure 2a. We want the metasurface to split the incident light equally into the ±1 diffraction orders for any incident angle θ_in_ within a 2θ_in_^max^ angular range. Such a broad-angle beam splitter can be used with a vertical-cavity surface-emitting laser (VCSEL) array and a microlens array as a dot projector to generate structured illumination useful for structured illumination microscopy,^45,46^ 3D endoscopy,^47,48^ and 3D sensing (*e.g.*, facial recognition,^49^ and motion detection^50^). The microlens array collimates the output from the VCSEL array, and the beam splitter increases the number of dots. VCSEL arrays are widely used for dot projectors due to their uniform intensity pattern, high power density, low cost, and simple packaging. However, existing beam splitters based on Dammann gratings^51,52^ or metasurfaces^53–55^ only operate on normal incident light and not the oblique incidence from the off-axis VCSEL units.

We let the metasurface be periodic with the width of one period being *W* = 24 μm, which couples transverse angular momenta *k*_*y*_ differing by integer multiples of 2π/*W*. Within the width *W*, we place *N* = 80 amorphous-silicon ridges (*n*_ridge_ = 3.70) with height *h* = 0.56 μm and varying widths and positions, sitting on a silica (*n*_sub_ = 1.45) substrate and surrounded by air (*n*_air_ = 1), as shown in Figure 2b,c. The ridge height ensures a sufficient 2π range of phase shifts when varying the ridge width (Section S2 and Figure S2). Since the desired response is symmetric, we let the structure be mirror symmetric with respect to a central plane at *y* = 0 (black dot-dashed line). The operating wavelength is λ = 940 nm, typical for VCSELs. The angular range is 2θ_in_^max^ = 60°, typical for dot projectors.

To inverse design this broad-angle beam splitter, we minimize the following FoM
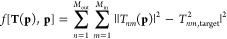
5where *T*_*nm*_ is the transmission coefficient from the *m-*th incident angle to the *n*-th outgoing angle. Here, **p** = {*p*_*k*_} = {*y*_1_, ..., *y*_*N*_} parametrize the two edge positions of the *N*/2 ridges within half a period of the metasurface (encircled by the red box in Figure 2b), with {*y*_*N*+1_, ..., *y*_2*N*_} = −{*y*_*N*_, ..., *y*_1_} based on symmetry. The target transmission is *T*_*nm*,target_^2^ = 0.5 at the ±1 diffraction orders and *T*_*nm*,target_^2^ = 0 otherwise. Equation 5 sums over all incident angles within the angular range of interest [*i.e.*, |*k*_*y*_^*m*^| < (2π/λ) sin θ_in_^max^ with 2π/*W* spacing] and all outgoing angles. With *W* = 24 μm, we have *M*_in_ = 25 input channels within the 60° angular range and *M*_out_ = 51 output channels. For this specific FoM, ∂*f*/∂*p*_*k*_ = 0 and ∂*f*/∂*T*_*nm*_ = 2(|*T*_*nm*_|^2^ – *T*_*nm*,target_^2^)*T*_nm_^*^ with * standing for complex conjugation. With a discretization grid size of Δ*x* = λ/40, we have nnz(**A**) ≈ 3.3 × 10^5^ and *M*_*p*_ = 3840. The APF-based objective-plus-gradient evaluation with *N*_sub_ = 3 takes 1.6 s while using 0.16 GiB of memory when running on one core; an objective-only evaluation takes 0.3 s. To validate that there is no mistake in our derivation and implementation, we show this in Figure S3 that the gradient computed with APF agrees with a brute-force finite-difference evaluation of the FoM in eq 5.

To minimize the FoM, we used gradient-based algorithms to update the optimization variables **p**. The optimizations stop when the change in *f* is less than *f*_tol_^abs^ = 10^–4^. After comparing the backtracking gradient descent, an interior-point method, the method of moving asymptotes, and the sequential least-squares quadratic programming (SLSQP) method (Section S4 and Figure S4), we choose the SLSQP^56^ implemented in the open-source package NLopt^57^ since it typically converges the fastest and often to a lower local minimum. During the optimization, the separation between neighboring edges (both the ridge width and the spacing between ridges) is constrained to be at least 40 nm to ensure fabrication feasibility.

**Figure 3:**
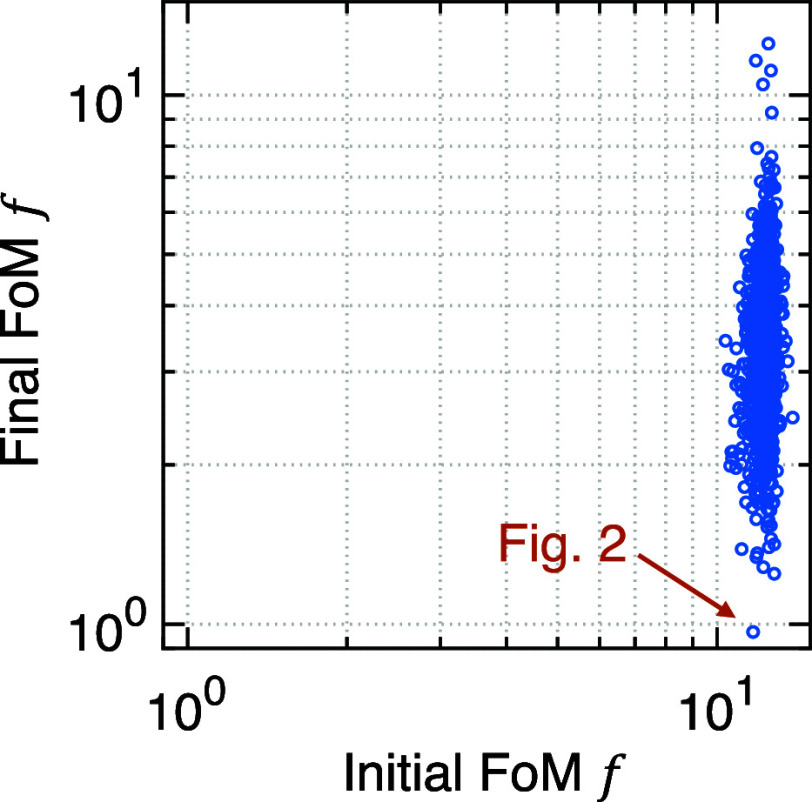
Final FoM and the initial FoM for 1000 randomly generated initial configurations.

We find that randomly sampled configurations of the parameter **p** have poor performance with the FoM narrowly distributed between 10 and 20, but the SLSQP optimization using those configurations as the initial guess leads to a wide distribution of the optimized FoM (Figure 3). Since this inverse-design problem is nonconvex, there is a sensitive dependence on the initial guess. To find a good final design, we run SLSQP optimizations with 1000 different initial guesses.

Figure 2c,d shows the initial configuration, the final configuration, their corresponding transmission matrices, and the evolution of FoM for the best case among these optimizations. The optimized metasurface exhibits uniform and near-perfect beam splitting for all incident angles within the 60° angular range of interest. Here, |*T*_*nm*_|^2^ averages to 0.4 at the ±1 diffraction orders and to 0.003 away from these angles. Video S1 shows how the configuration and the transmission matrix evolve, and Table S2 lists the parameters of the final configuration.

In Sections S6 and S7 and Figures S6–S8 of the Supporting Information, we consider (1) optimizations where we do not impose a mirror symmetry in the structure and (2) optimizations where the center positions of the ridges are fixed on a periodic lattice to mimic a unit-cell-based design (with ridge widths being the only optimization variables). The no-mirror-symmetry configuration has a larger-than-necessary design space, which is harder to search, and it yields a less optimal result while requiring more iterations. The fixed-center configuration has a smaller design space, leading to fewer iterations but also less optimal result. Note that in these instances, increasing or reducing the number of design variables *N*_*p*_ does not change *M*_*p*_ (the number of pixels modified by those design variables), so the computing time and memory usage per iteration do not change.

## Conclusions

With traditional adjoint methods, the computing cost grows with the number of input channels. APF eliminates that channel-number dependence, and the main factor becomes *M*_*p*_, the number of pixels modified by the design variables. When , the computing cost is independent of both the number of channels and the number of design variables. In situations with , one can interpolate the response at nearby pixels to reduce *M*_*p*_ and/or increase the number *N*_sub_ of divisions, as shown in Figure 1d. If one chooses , the total computing time (or the number of computing nodes) will grow linearly with *M*_*p*_, and the memory usage will be independent of *M*_*p*_. Instead of the matrix division employed here, future work may also explore computing the Schur complement of a rectangular augmented matrix to avoid computing unnecessary matrix **U**^T^**A**^–1^**U**.

The APF-based multichannel gradient computation formalism here also applies in 3D, though with a higher computing cost. For a 3D system with thickness *h* = 0.56 μm, lateral area *a* = *W*^2^ = 16*h*^2^, discretized with grid size Δ*x* = λ/40 at λ = 940 nm, a 3D vectorial APF computation^58^ for a multichannel objective evaluation takes 8.3 min using 22 GiB of memory when running on an Intel Xeon 2640v4 node with multithreading; the computing time and memory usage scale as  and , respectively, for such geometry. Larger areas can be stitched together as in refs (59−62). The scaling with the number of design parameters is governed by *M*_*p*_ and is the same as that in 2D.

Computer-automated design and discovery unlock numerous possibilities, and the formalism proposed here can be an enabling element for inverse design on a wide range of multichannel optical systems mentioned in the Introduction section. Advances in the computation method, together with open-source codes, can usher in the next generation of photonic devices.

## Data Availability

All data needed to evaluate the conclusions in this study are presented in the paper and Supporting Information. The code is available on GitHub.^44^

## Supporting Information Available

Benchmarks on the gradient computation using the APF and a conventional adjoint method; amplitude and phase maps of the meta-atoms in a periodic configuration; comparison of the gradient obtained from APF and finite difference; comparison of different optimization algorithms; and broad-angle beam splitters using metasurfaces without mirror symmetry and metasurfaces with fixed ridge center positions (PDF)
Animation of how the metasurface and its transmission matrix evolve as the optimization progresses (MP4)

## Notes

An initial draft was deposited in the preprint service arXiv as Li, S.; Lin, H.-C.; and Hsu, C. W. Fast Multichannel Inverse Design through Augmented Partial Factorization. *arXiv***2023**, arXiv:2306.09257. https://arxiv.org/abs/2306.09257 (accessed June 28, 2023).
