# Supplementary material for: Fast Multichannel Inverse Design through Augmented Partial Factorization
Source: ACS Photonics. 2024 Jan 4;11(2):378–84. doi: 10.1021/acsphotonics.3c00911 (PMC10885196; doi:10.1021/acsphotonics.3c00911)
Supplement: Supplementary file 1 — ph3c00911_si_001.pdf [file ph3c00911_si_001.pdf]

Fast Multi-Channel Inverse Design through Augmented Partial Factorization: Supporting Information

Shiyu Li, Ho-Chun Lin, and Chia Wei Hsu*
Ming Hsieh Department of Electrical and Computer Engineering,
University of Southern California, Los Angeles, California 90089, USA

This document provides supporting information to "Fast multi-channel inverse design through augmented partial factorization". It consists of seven sections. Section 1 provides benchmarks on the gradient computation using the augmented partial factorization (APF) and a conventional adjoint method. Section 2 gives the amplitude and phase maps of the meta-atoms in a periodic configuration. Section 3 compares the gradient obtained from APF and from finite difference. Section 4 compares the performance of different optimization algorithms for designing a broad-angle beam splitter. In Sec. 5, we use animation to show how the metasurface and its transmission matrix evolve as the optimization progresses. In Sec. 6–7, we show the design of broad-angle beam splitters using metasurfaces without mirror symmetry and metasurfaces with fixed ridge center positions.

Number of pages: 7
Number of figures: 8
Number of tables: 2

CONTENTS

1. Benchmarks on gradient computation S1

2. Meta-atoms of the metasurface S3

3. Gradient validation S3

4. Comparison of optimization algorithms S3

5. Evolution of the optimization (animation) S5

6. Optimization without mirror symmetry S5

7. Optimization with fixed ridge centers S6

References S7

1. BENCHMARKS ON GRADIENT COMPUTATION

As an example, here we compare the computing time and memory usage of f only and f-plus-$\boldsymbol{\nabla}_{\mathbf{p}} f$ for a 1-mm-wide metasurface, using the augmented partial factorization (APF)[1] and a conventional adjoint method. The metasurface (1200λ wide) contains 1200 amorphous-silicon ridges ($n_{\rm ridge} = 3.70$) with height 0.6λ sitting on a silica substrate ($n_{\rm sub} = 1.45$); it has mirror symmetry with respect to the central plane such that the $N_p = 1200$ edge positions of ridges on the left-half side of the metasurface can be changed independently. We evaluate the full-wave responses with $M_{\rm in} = M_{\rm out} = 2W/\lambda = 2400$ inputs and outputs using the open-source software MESTI[2].

For the APF method, we build matrices $\mathbf{B}$ and $\mathbf{C}$ using channels from both sides of the metasurface and retrieve the response of the desirable channels after the partial factorization to take advantage of the symmetry of the augmented matrix $\mathbf{K} = [\mathbf{A}, \mathbf{B}; \mathbf{C}, \mathbf{D}]$ and reduce the computing time and memory usage. Note that here ${\rm nnz}(\mathbf{B}) \approx 2.3 \times 10^8$ exceeds ${\rm nnz}(\mathbf{A}) \approx 1.6 \times 10^7$, so we compress $\mathbf{B}$ and $\mathbf{C}$ to reduce the number of nonzero elements such that ${\rm nnz}(\mathbf{B})$

* cwhsu@usc.edu

TABLE S1. **Computing time and memory usage for a 1-mm-wide metasurface**

$N = 1200$ ridges<br>2399 input angles, 2399 output angles				
	f only		f & $\nabla_{\mathbf{p}} f$	
Method	APF	Conventional	APF $N_{\text{sub}} = 15$	Conventional adjoint
Computing time (minute)				
Build	0.03	58	0.4	113
Analyze	0.05	127	0.8	249
Factorize	0.24	395	5.7	788
Solve	0	95	0	192
Decompress	0.01	0	0.3	0
Post-process	0.0	0.0	2.3	12
Total	0.33	675	9.5	1354
Memory usage (GiB)				
	4	10	7	10

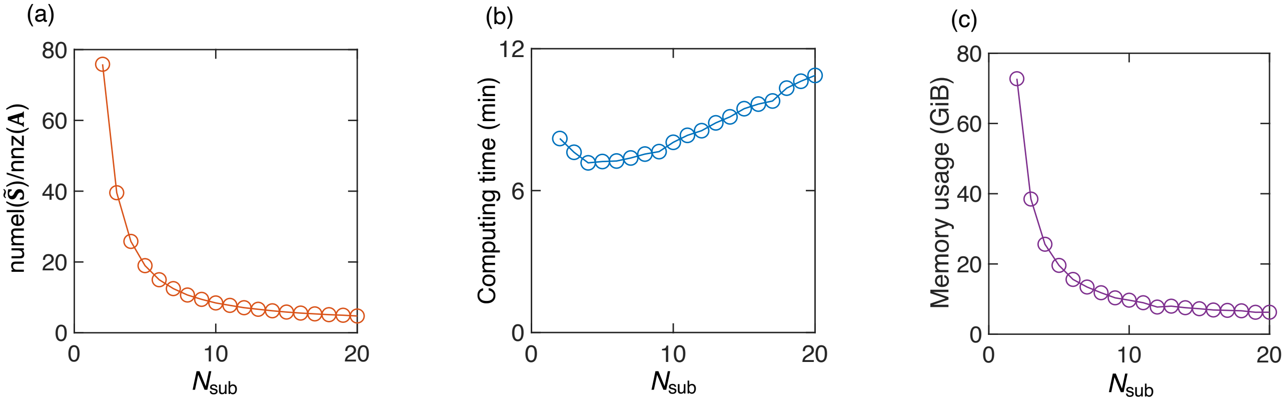

FIG. S1. (a) The size of matrix $\tilde{\mathbf{S}}$ compared to the number of nonzero elements in matrix $\mathbf{A}$ when one objective-plus-gradient APF computation is divided into N_{sub} sub-ones for a 1-mm-wide metasurface. The (b) total computing time and (c) memory usage as a function of N_{sub}.

$< \mathrm{nnz}(\mathbf{A})$ and the computational cost does not grow with M_{in}[1]. Specifically, we pad 480 extra channels to the input/output profiles, weight them by the Hann window function, Fourier transform them to the spatial domain, and then truncate with a 3λ window. After performing the partial factorization, we decompress by undoing the above process, resulting in a negligible error of $\sim 10^{-4}$ from compression.

For the conventional method when only f is needed, we compute $\mathbf{A}^{-1}\mathbf{B}_m$ and project it with $\mathbf{C}$, and repeat this for each input m. To compute the gradient using the conventional adjoint method, we compute $\mathbf{A}^{-1}\mathbf{B}_m$ (the forward simulation) and then $\mathbf{C}_m^{\text{adj}}\mathbf{A}^{-1}$ (the adjoint simulation) for each input m, with

$$\mathbf{C}_m^{\text{adj}} = \sum_{n=1}^{M_{\text{out}}} \frac{\partial f}{\partial S_{nm}} \mathbf{C}_n. \tag{1}$$

By substituting $\partial \mathbf{S}/\partial p_k = -\mathbf{C}\mathbf{A}^{-1}(\partial \mathbf{A}/\partial p_k)\mathbf{A}^{-1}\mathbf{B}$ into Eq. (1) of the main text, we can see that

$$\frac{df}{dp_k} = \frac{\partial f}{\partial p_k} - \sum_{m=1}^{M_{\text{in}}} 2\mathrm{Re}\left[\left(\mathbf{C}_m^{\text{adj}}\mathbf{A}^{-1}\right)\frac{\partial \mathbf{A}}{\partial p_k}\left(\mathbf{A}^{-1}\mathbf{B}_m\right)\right]. \tag{2}$$

So, for this conventional adjoint method, we build the gradient using a loop over the M_{in} inputs, with M_{in} forward simulations and M_{in} adjoint simulations.

We have made our gradient computation code (both with APF and with the conventional adjoint method) open-

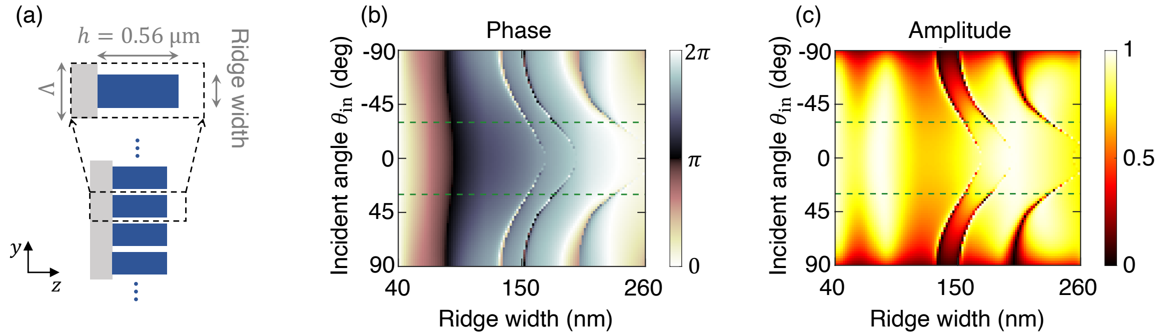

FIG. S2. (a) Schematic structure of a periodic array of unit cells. (b) Phase and (c) amplitude maps of the zeroth-order transmission coefficient for different ridge widths (from 40 nm to 260 nm) and incident angles. The green dashed lines mark the angular range of interest, $\theta_{\rm in} \in [-30^\circ, 30^\circ]$.

source[3]. All computations are performed using a single core on an Intel Xeon 2640v4 node. Each case is computed 10 times. Table S1 shows the average computing time and the maximal memory usage after subtracting the 0.9 GiB memory used by MATLAB R2022a. The time needed to obtain f and $\nabla_{\mathbf{p}} f$ from the simulations is counted as part of post-processing. For the f-only case, APF outperforms the conventional method by $\sim$2000 fold in speed and $\sim$2.5 fold in memory, with the memory usage difference coming mainly from the compression of $\mathbf{B}$ and $\mathbf{C}$. For the gradient computation, APF still outperforms the conventional adjoint method with a $\sim$150 fold speed-up and a $\sim$30% reduction in memory usage. Here, the APF computation is separated into $N_{\rm sub} = 15$ ones. Figure S1 further shows the $N_{\rm sub}$ dependence.

2. META-ATOMS OF THE METASURFACE

Figure S2 shows that periodic ridges with periodicity $\Lambda = 300$ nm, height $h = 0.56$ µm, and varying ridge widths can provide a 2π range of phase shift with high transmission over the 60° angular range of interest. These unit-cell simulations are also performed with MESTI[2], adopting a periodic boundary in y. Note that these unit-cell simulations are only used to choose an appropriate ridge height h. Except for Sec. 7, the inverse design we consider is not restricted to any unit-cell configurations.

3. GRADIENT VALIDATION

To validate the gradient derivation and implementation, we compare the gradient computed with APF and an approximate gradient evaluated through finite difference. We consider the metasurface with $N = 80$ ridges described in Fig. 2 of the main text, with the FoM f defined in Eq. (5). The transmission matrix $\mathbf{T}$ and FoM f are evaluated at $\mathbf{p}_0$ and $\mathbf{p}_0 + \Delta\mathbf{p}$, where $\Delta\mathbf{p}$ is a random increment of design parameters. Taylor expansion shows that the l^2-norm of the matrix difference, $||\mathbf{\Delta T} - \nabla_{\mathbf{p}}\mathbf{T}(\mathbf{p}_0 + \Delta\mathbf{p}/2)\cdot\Delta\mathbf{p}||$ with $\mathbf{\Delta T} = \mathbf{T}(\mathbf{p}_0 + \Delta\mathbf{p}) - \mathbf{T}(\mathbf{p}_0)$, should scale as $\mathcal{O}(||\Delta\mathbf{p}||^3)$ when $||\Delta\mathbf{p}||$ is small if the gradient $\nabla_{\mathbf{p}}\mathbf{T}(\mathbf{p}_0 + \Delta\mathbf{p}/2)$ is correct. The same is true for the FoM difference $|\Delta f - \nabla_{\mathbf{p}} f(\mathbf{p}_0 + \Delta\mathbf{p}/2)\cdot\Delta\mathbf{p}|$ with $\Delta f = f(\mathbf{p}_0 + \Delta\mathbf{p}) - f(\mathbf{p}_0)$. Figure S3 shows that we indeed observe these scalings. We also confirmed that APF and the conventional adjoint method yield the same gradient.

4. COMPARISON OF OPTIMIZATION ALGORITHMS

There are many well-developed gradient-based algorithms. Figure S4 compares how the FoM evolves using four different algorithms, starting from the same initial guess. All of them impose the separation between neighboring edges (both the ridge width and the spacing between ridges) to be at least 40 nm through inequality constraints.

The simplest method is a gradient descent: update the parameter vector $\mathbf{p}$ along the opposite direction of $\nabla_{\mathbf{p}} f$ as $\mathbf{p}_{n+1} = \mathbf{p}_n - \gamma_n \nabla_{\mathbf{p}} f(\mathbf{p}_n)$. Here, γ_n sets the step size (also called the "learning rate" in the machine learning literature) at the n-th iteration. The choice of the learning rate is important. Here, we choose γ_n with the "backtracking line search" method[4,5] to avoid overshooting (when γ_n is too large) or slowing down the convergence (when γ_n is too small). At each iteration n, we start with a learning rate of $\gamma_n = \min[\gamma_{\rm max}, 10\gamma_{n-1}]$ and then iteratively shrink the

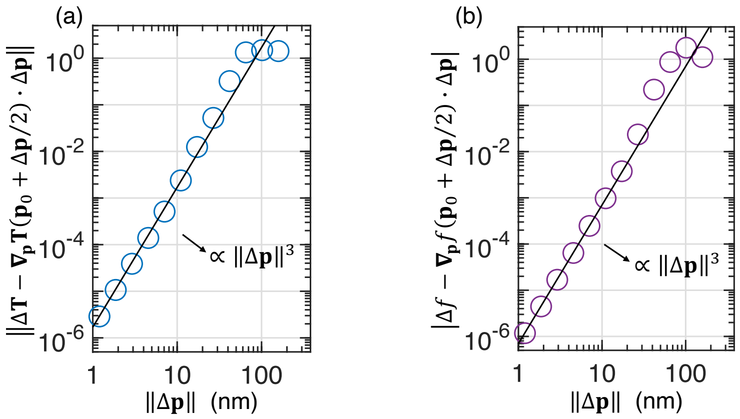

FIG. S3. Log-log plots of (a) the matrix difference $||\Delta\mathbf{T} - \nabla_\mathbf{p}\mathbf{T}(\mathbf{p}_0 + \Delta\mathbf{p}/2)\cdot\Delta\mathbf{p}||$ and (b) the FoM difference $|\Delta f - \nabla_\mathbf{p} f(\mathbf{p}_0 + \Delta\mathbf{p}/2)\cdot\Delta\mathbf{p}|$ as a function of the step size $||\Delta\mathbf{p}||$. Here, $\Delta\mathbf{T} = \mathbf{T}(\mathbf{p}_0 + \Delta\mathbf{p}) - \mathbf{T}(\mathbf{p}_0)$ and $\Delta f = f(\mathbf{p}_0 + \Delta\mathbf{p}) - f(\mathbf{p}_0)$. The solid black lines have a slope of 3, showing the expected $\mathcal{O}(||\Delta\mathbf{p}||^3)$ scaling for small $||\Delta\mathbf{p}||$.

learning rate by a factor of $\tau = 1/2$ until the Armijo condition[6] is satisfied:

$$f[\mathbf{p}_n - \gamma_n \nabla_\mathbf{p} f(\mathbf{p}_n)] \le f(\mathbf{p}_n) - c_1\gamma_n[\nabla_\mathbf{p} f(\mathbf{p}_n)]^\mathrm{T}\nabla_\mathbf{p} f(\mathbf{p}_n), \tag{3}$$

with $c_1 = 10^{-2}$. Here, $\nabla_\mathbf{p} f$ is expressed as a column vector, and $^\mathrm{T}$ denotes vector transpose. For a random initial guess, $|df/dp_k|$ is on the order of 30 µm^{-1}, and we set $\gamma_{\max} = 2\times10^{-4}$ µm^2. The intermediate backtracking line search steps only evaluate the FoM, not the gradient. At the same time, We make sure that the optimization variables are within the feasible region upon each update and shrink the update size if not.

In addition to the backtracking gradient descent, we also consider the interior point method[7–9] implemented in the `fmincon` function in MATLAB and the method of moving asymptotes (MMA)[10] and the sequential least-squares quadratic programming (SLSQP) method[11] implemented in the open-source package NLopt[12].

As shown in Fig. S4, SLSQP converges faster and to a lower level among the four algorithms. Therefore, we adopt SLSQP for all optimizations in this work.

The backtracking gradient descent and SLSQP use intermediate steps that evaluate f without evaluating $\nabla_\mathbf{p} f$. Since the gradient evaluation takes more resources, we only count the number of gradient evaluations in the x axis of Fig. S4. The interior point method and MMA both evaluate $\nabla_\mathbf{p} f$ at all steps.

The line search of our gradient descent implementation ensures that the FoM moves strictly downhill at every iteration when the intermediate line search steps are excluded. The other three methods have a more flexible iteration scheme that allows overshooting, so their FoM can occasionally move upward.

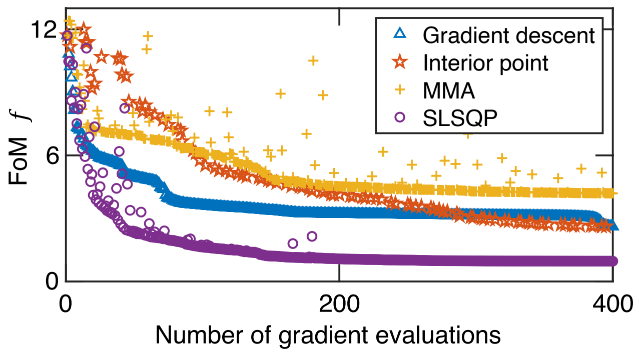

FIG. S4. The evolution of the FoM starting from the same initial guess and using four different algorithms.

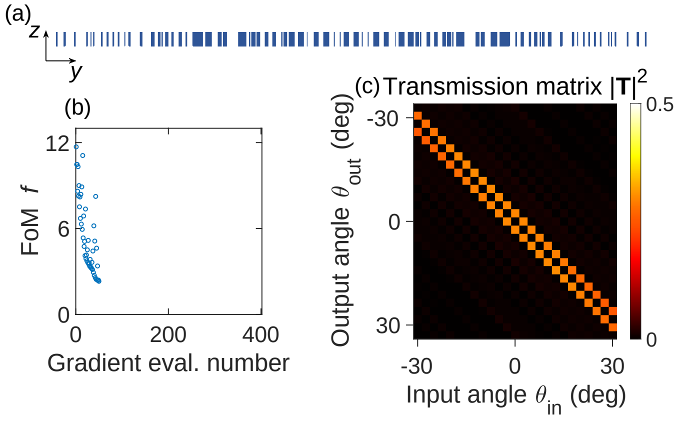

FIG. S5. One frame (at the 50-th gradient evaluation) of Supplementary Video 1, which shows how (a) the metasurface structure, (b) the FoM, and (c) the transmission matrix evolve as the optimization progresses.

TABLE S2. **Edge positions of the optimized metasurface (in micron)**

$\{y_1, \cdots, y_8\}$	-11.863, -11.778, -11.497, -11.413, -11.131, -11.047, -10.791, -10.708
$\{y_9, \cdots, y_{16}\}$	-10.503, -10.463, -10.423, -10.357, -10.111, -10.026, -9.769, -9.695
$\{y_{17}, \cdots, y_{24}\}$	-9.581, -9.512, -9.300, -9.221, -9.130, -9.086, -8.927, -8.843
$\{y_{25}, \cdots, y_{32}\}$	-8.557, -8.472, -8.183, -8.097, -7.806, -7.730, -7.674, -7.632
$\{y_{33}, \cdots, y_{40}\}$	-7.489, -7.361, -7.203, -7.111, -6.952, -6.814, -6.648, -6.560
$\{y_{41}, \cdots, y_{48}\}$	-6.317, -5.891, -5.753, -5.530, -5.354, -5.151, -5.111, -4.963
$\{y_{49}, \cdots, y_{56}\}$	-4.577, -4.267, -4.103, -4.047, -4.007, -3.796, -3.741, -3.633
$\{y_{57}, \cdots, y_{64}\}$	-3.593, -3.452, -3.170, -3.007, -2.956, -2.916, -2.701, -2.575
$\{y_{65}, \cdots, y_{72}\}$	-2.529, -2.306, -2.137, -1.905, -1.678, -1.635, -1.432, -1.249
$\{y_{73}, \cdots, y_{80}\}$	-1.179, -0.964, -0.695, -0.655, -0.443, -0.400, -0.322, -0.094

5. EVOLUTION OF THE OPTIMIZATION (ANIMATION)

Supplementary Video 1 shows how the metasurface structure, its transmission matrix $|\mathbf{T}|^2$, and the FoM evolve as the optimization progresses. Figure S5 provides the animation caption and shows one frame of the animation.

The edge positions $\mathbf{p} = \{p_k\} = \{y_1, \cdots, y_{80}\}$ for one half of the optimized 80-ridge metasurface are listed in Tab. S2. The other half is symmetric, $\{y_{81}, \cdots, y_{160}\} = -\{y_{80}, \cdots, y_1\}$, so they are not listed.

6. OPTIMIZATION WITHOUT MIRROR SYMMETRY

Figure 2 of the main text shows the optimized broad-angle metasurface beam splitter with mirror symmetry. Here, we show optimization results without imposing a mirror symmetry, where we update all edge positions $\{y_1, \cdots, y_{2N}\}$ independently. Like the symmetric setup, here we use the SLSQP algorithm and run 1000 instances of optimization with different initial guesses. Figure S6 shows the structures and their transmission matrices before and after optimization, for the best case among the 1000 initial guesses. It has $|T_{nm}|^2$ averaged to 0.3 at the ± 1 diffraction orders over the 60° angular range.

Figure S7 summarizes the final FoM and the optimization time of the 1000 instances, for both metasurfaces with and without mirror symmetry. The without-mirror-symmetry scenario tends to yield a worse FoM and take longer, because its parameter space is unnecessarily large and contains more poor local minima.

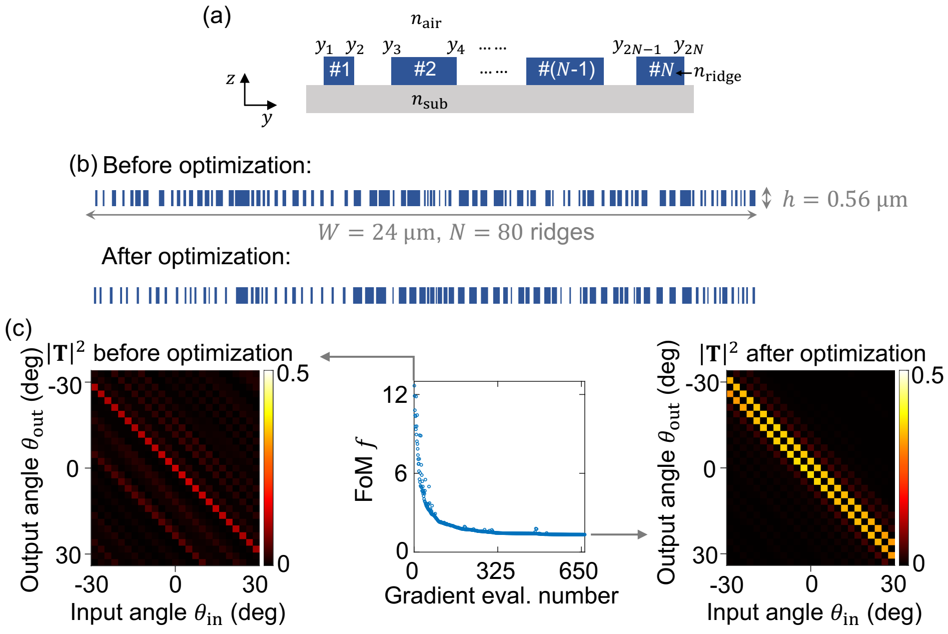

FIG. S6. Schematic of (a) a general metasurface without mirror symmetry. (b) Metasurfaces before and after optimization. (c) Evolution of the FoM f during optimization and the squared amplitude of transmission matrices before and after optimization, for the best case among 1000 random instances.

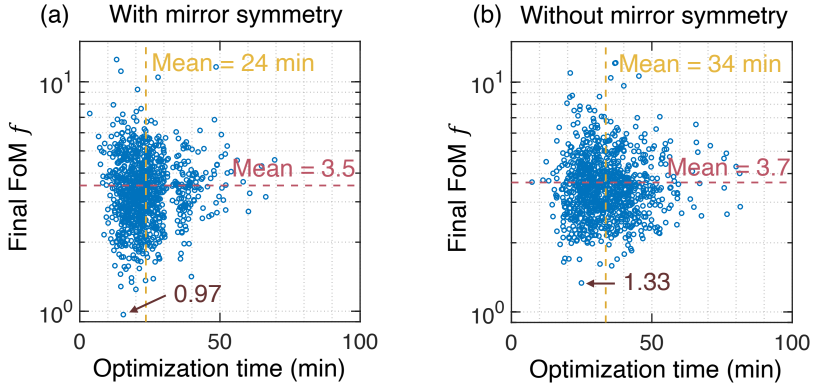

FIG. S7. The final FoM and the optimization time starting from 1000 random initial guesses, with dashed lines representing the average FoM (red) and the average optimization time (yellow), for metasurfaces (a) with mirror symmetry and (b) without mirror symmetry. The minimal FoM achieved over these 1000 optimizations is marked by an arrow.

7. OPTIMIZATION WITH FIXED RIDGE CENTERS

In Figs. 2–3 of the main text and in the above sections, we allow both the width and the center position of the ridges to change freely during the optimization; the structure is not tied to any periodic unit-cell configuration. To assess the performance gain from going beyond a unit-cell-based design, here we perform optimizations where the center positions of the ridges are fixed on a periodic lattice (with ridge widths being the only optimization variables). In this case, each ridge can be considered as one unit cell, though we still perform full-wave simulation of the entire metasurface (without the locally periodic approximation commonly used in unit-cell-based designs). Figure S8 shows the optimal results from 1000 instances of optimization. The metasurface is mirror symmetric with respect to its central plane (black dot-dashed line), and the periodicity of the unit cells is $\Lambda = 300$ nm. This best case has $|T_{nm}|^2$ averaged to 0.3 at the ± 1 diffraction orders over the 60° angular range with an optimal FoM of 1.31, which is less optimal compared to Fig. 2 of the main text due to its limited design space.

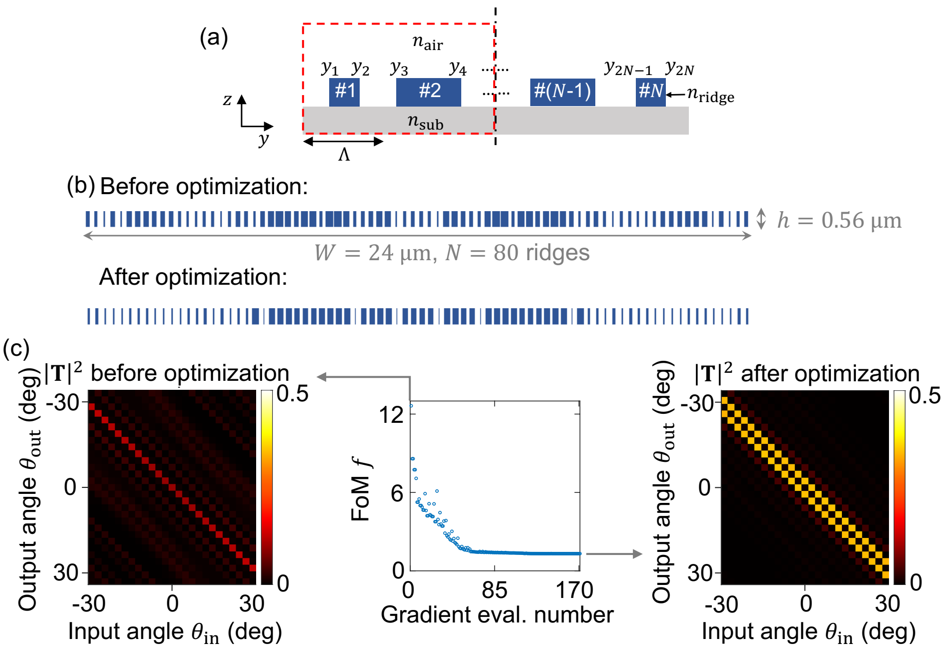

FIG. S8. (a) Schematic of a mirror-symmetric metasurface with the ridge centers fixed on a periodic lattice with periodicity $\Lambda = 300$ nm. Only the ridge widths are varied during optimization. (b) Metasurfaces before and after optimization. (c) Evolution of the FoM during optimization and the squared amplitude of the transmission matrices before and after optimization.

[1] H.-C. Lin, Z. Wang, and C. W. Hsu, Fast multi-source nanophotonic simulations using augmented partial factorization, Nat. Comput. Sci. **2**, 815 (2022).

[2] H.-C. Lin, Z. Wang, and C. W. Hsu, MESTI, `https://github.com/complexphoton/MESTI.m` (visited on 09/26/2022).

[3] S. Li and C. W. Hsu, APF Inverse Design, `https://github.com/complexphoton/APF_inverse_design` (visited on 06/16/2023).

[4] J. Nocedal and S. J. Wright, Line search methods, in *Numerical Optimization* (Springer, 2006).

[5] T. T. Truong and H.-T. Nguyen, Backtracking gradient descent method and some applications in large scale optimization. part 2: Algorithms and experiments, Appl. Math. Optim. **84**, 2557 (2021).

[6] L. Armijo, Minimization of functions having lipschitz continuous first partial derivatives, Pac. J. Math. **16**, 1 (1966).

[7] R. H. Byrd, M. E. Hribar, and J. Nocedal, An interior point algorithm for large-scale nonlinear programming, SIAM J. Optim. **9**, 877 (1999).

[8] R. H. Byrd, J. C. Gilbert, and J. Nocedal, A trust region method based on interior point techniques for nonlinear programming, Math. Program. **89**, 149 (2000).

[9] R. A. Waltz, J. L. Morales, J. Nocedal, and D. Orban, An interior algorithm for nonlinear optimization that combines line search and trust region steps, Math. Program. **107**, 391 (2006).

[10] K. Svanberg, A class of globally convergent optimization methods based on conservative convex separable approximations, SIAM J. Optim. **12**, 555 (2002).

[11] D. Kraft, Algorithm 733: TOMP–Fortran modules for optimal control calculations, ACM Trans. Math. Softw. **20**, 262 (1994).

[12] S. G. Johnson, The NLopt Nonlinear-Optimization Package, `https://github.com/stevengj/nlopt` (visited on 09/26/2022).
